# Logistical, cultural, and structural barriers to immediate neonatal care and neonatal resuscitation in Bihar, India

**DOI:** 10.1186/s12884-018-2017-5

**Published:** 2018-09-29

**Authors:** Brennan Vail, Melissa C. Morgan, Jessica Dyer, Amelia Christmas, Susanna R. Cohen, Megha Joshi, Aboli Gore, Tanmay Mahapatra, Dilys M. Walker

**Affiliations:** 1 Department of Pediatrics, University of California San Francisco, 550 16th Street, 4th Floor, Box 0110, San Francisco, CA 94143 USA; 2 Institute for Global Health Sciences, University of California San Francisco, 550 16th Street, 3rd Floor, Box 1224, San Francisco, CA 94158 USA; 3 Maternal, Adolescent, Reproductive, and Child Health Centre, London School of Hygiene and Tropical Medicine, Keppel Street, London, WC1E 7HT UK; 4 PRONTO International, 1820 E. Thomas Street APT 16, Seattle, WA USA; 5 PRONTO International, State RMNCH+A Unit, C-16 Krishi Nagar, A.G. Colony, Patna, Bihar 80002 India; 6 College of Nursing, University of Utah, 10 South 2000 East, Salt Lake City, UT 84112 USA; 7 CARE India Solutions for Sustainable Development, Bihar Technical Support Unit, House No.14, Patliputra Colony, Patna, Bihar 800013 India; 8 CARE India Solutions for Sustainable Development, Bihar Technical Support Unit, House No.14, Patliputra Colony, Patna, Bihar 800013 India; 9 CARE India Solutions for Sustainable Development, Bihar Technical Support Unit, House No.14, Patliputra Colony, Patna, Bihar 800013 India; 10 Department of Obstetrics and Gynecology and Reproductive Services, University of California San Francisco, 1001 Potrero Ave, San Francisco, CA 94110 USA

**Keywords:** Bihar, India, Neonatal resuscitation, Evidence-based practices, Barriers to care

## Abstract

**Background:**

In India, the neonatal mortality rate is nearly double the Sustainable Development Goal target with more than half of neonatal deaths occurring in only four states, one of which is Bihar. Evaluations of immediate neonatal care and neonatal resuscitation skills in Bihar have demonstrated a need for significant improvement. However, barriers to evidence based practices in clinical care remain incompletely characterized.

**Methods:**

To better understand such barriers, semi-structured interviews were conducted with 18 nurses who participated as mentors in the AMANAT maternal and child health quality improvement project, implemented by CARE India and the Government of Bihar. Nurse-mentors worked in primary health centers throughout Bihar facilitating PRONTO International emergency obstetric and neonatal simulations for nurse-mentees in addition to providing direct supervision of clinical care. Interviews focused on mentors’ perceptions of barriers to evidence based practices in immediate neonatal care and neonatal resuscitation faced by mentees employed at Bihar’s rural primary health centers. Data was analyzed using the thematic content approach.

**Results:**

Mentors identified numerous interacting logistical, cultural, and structural barriers to care. Logistical barriers included poor facility layout, supply issues, human resource shortages, and problems with the local referral system. Cultural barriers included norms such as male infant preference, traditional clinical practices, hierarchy in the labor room, and interpersonal relations amongst staff as well as with patients’ relatives. Poverty was described as an overarching structural barrier.

**Conclusion:**

Interacting logistical, cultural and structural barriers affect all aspects of immediate neonatal care and resuscitation in Bihar. These barriers must be addressed in any intervention focused on improving providers’ clinical skills. Strategic local partnerships are vital to addressing such barriers and to contextualizing skills-based trainings developed in Western contexts to achieve the desired impact of reducing neonatal mortality.

## Background

Between 1990 and 2015, low- and middle-income countries (LMICs) achieved a 53% reduction in mortality among children less than 5 years of age. Although a significant achievement, this improvement fell short of the Millennium Development Goal target of 67% [1]. The relative lack of improvement in neonatal survival, defined as survival through the first 28 days of life, explains, in part, why this goal was not reached. Neonatal deaths accounted for approximately 43% of deaths among children under age five globally in 2016 [2]. In response, the Sustainable Development Goals (SDG) have placed renewed emphasis on neonatal survival and set a target neonatal mortality rate (NMR) of 12 per 1000 live births in all countries by 2030 [3].

In India, substantial improvement in neonatal care will be required to meet this goal, as the countrywide NMR in 2016 was 21.8 [2]. Success will likely be contingent on understanding and addressing the variations in neonatal mortality across the 29 Indian states, as more than half of neonatal deaths occur in only four states- Bihar, Uttar Pradesh, Madhya Pradesh, and Rajasthan [4]. Additionally, the NMR in rural India is more than double that of urban India, and the NMR in the poorest 20% of the population is more than double that in the richest 20% [4]. One-third of neonatal deaths in India occur within 24 h after birth, and the leading causes of neonatal death are preterm birth (< 37 completed weeks of gestation), birth asphyxia, and infection [4]. Therefore, interventions aimed at improving the immediate care of neonates born to families living in rural Bihar, Uttar Pradesh, Madhya Pradesh, and Rajasthan may have the greatest impact on reducing the NMR in India.

In 2014, the World Health Organization developed the “Every Newborn Action Plan” to guide such interventions. This plan calls for research that explores barriers to evidence-based practices (EBP) in essential newborn care and neonatal resuscitation (NR) [5, 6]. Multi-country analyses of barriers have identified numerous bottlenecks to care in LMICs related to leadership/governance, financing, workforce availability and skill, essential commodities, delivery of care, health information systems, and community partnerships [7–11]. A similar analysis in India cited leadership/governance, human resources, and health information systems as the most significant national barriers [4]. However, given the previously described variations in NMR across Indian states, a more focused evaluation of areas with the highest burden of neonatal mortality is needed to guide targeted interventions in these regions.

The state of Bihar, located in northeastern India, was found to be the poorest region in all of South Asia (including Afghanistan, Bangladesh, Bhutan, India, Maldives, Nepal, and Pakistan) in 2016 [12]. Based on the most recent census, Bihar had the highest crude birth rate in all of India at 27.7 and a NMR of 28 [13], with significant under-reporting likely. The majority of the basic obstetric and neonatal care in Bihar is provided through primary health centers (PHCs), each of which serve a population of approximately 190,000 (number based on monitoring and evaluation data from CARE India [14]) and are often poorly equipped for neonatal care [15]. Obstetric and newborn care at PHCs are largely provided by nurses with an Auxiliary Nurse Midwife (ANM) qualification and occasionally by nurses with a General Nursing and Midwifery (GNM) qualification, which entail two and three and a half years of training respectively after completion of secondary school [16]. These nurses frequently lack adequate training and skills in basic NR [15]. Nevertheless, pediatricians are few in number and only available at higher levels of care [17].

Despite the many potential barriers exposed by demographic and health system data, to the best of our knowledge, a focused analysis of barriers to EBP in immediate neonatal care and NR in Bihar has not been conducted. This information is needed to inform and improve the effectiveness of ongoing and future interventions in Bihar. One such intervention is *Apatkaleen Matritva evam Navjat Tatparta (*AMANAT), a maternal and child health quality improvement program with a mentorship model of clinical instruction, implemented by CARE India and the Government of Bihar [14, 18, 19]. AMANAT mentors are nurses with a 4-year bachelor’s degree recruited from across India and mentees were ANMs or GNMs working in PHCs throughout Bihar. Within the AMANAT intervention, PRONTO International trained mentors to teach emergency obstetric and neonatal management to ANM/GNM mentees using simulation [20]. Ongoing evaluations of PRONTO training in Bihar have demonstrated that nurse mentees’ skills in NR lag behind their skill acquisition in obstetric emergencies [21]. In response, this manuscript aims to characterize the logistical, cultural, and structural barriers to the use of EBPs in immediate neonatal care, defined as care required during the immediate transition to post-natal life, and NR.

## Methods

### Procedures

A semi-structured interview guide was developed and informed by initial evaluations of PRONTO simulation training in Bihar [21], demographic and health systems data from Bihar [17, 22], and multi-country bottleneck analyses [7–11]. The interview guide employed open-ended questions about potential barriers while allowing the interviewer flexibility to ask additional questions on emerging themes. Pilot testing of the interview guide was conducted with a former AMANAT mentor to identify and revise unclear questions. Interviews were conducted by one lead investigator in English; all interviewees spoke fluent English. A local, Hindi-speaking member of the PRONTO International team arranged and attended interviews. To ensure anonymity and confidentiality, interviews were conducted in a private room at the PHC where the interviewees worked and no personal identifier was linked with the data. If an interview was conducted outside of working hours or if private space was unavailable at a PHC, the interview was conducted in a private space near the PHC. Interview duration ranged from 45 to 75 min.

### Recruitment and sampling

Interviews were conducted in January 2017. At that time, there were 40 nurse mentors working in pairs at PHCs throughout Bihar. In order to capture the broadest possible range of experiences, one mentor from each pair was selected for interview according to the following eligibility criteria: 1) working as an AMANAT mentor at the time of interview and 2) had participated in two phases of the AMANAT intervention (equivalent to 16 months of work at eight different PHCs). Two of the 20 mentors selected were unable to participate due to illness and personal travel. In total, 18 interviews were conducted with mentors who had cumulative experience at approximately 144 PHCs. With this data saturation was reached and interviews were stopped.

### Data analysis

Audio recordings of interviews were transcribed by the interviewer. Data were analyzed using the thematic content approach [23, 24]. This method of analysis was chosen as multi-country analyses of barriers to care [7–11] and demographic and health systems data from Bihar [17, 22] were available to inform the initial approach to analysis. Further, our goal was to identify and synthesize themes within our data rather than develop a novel theory. The analysis was conducted in four steps consistent with Braun and Clarke’s proposed method for thematic analysis [25]: 1) data familiarization, 2) identifying codes and themes, 3) developing a coding scheme and applying it to the data, and 4) organizing codes and themes. Two interviews were randomly selected for double coding by another lead investigator to ensure consistency in identification of key themes. Qualitative analysis was conducted manually without the use of software.

## Results

Demographic data from the 18 interviewees are presented in Table 1. All interviewees had a 4-year Bachelor’s degree in nursing, but none were originally from Bihar.

**Table 1:** Characteristics of Participants (*N* = 18)

Mentor Characteristics	n	Mean (95% CI)
Age in Years	16	25.5 (24.5–26.5)
Cumulative Years of Nursing Experience	16	3.0 (2.3–3.8)
		**n (%)**
Possesses a Bachelors Degree in Nursing	18	18 (100%)
Indian Zonal Council of Original Residence	18	
North		6 (33.3%)
South		4 (22.2%)
East		3 (16.7%)
West		3 (16.7%)
Central		2 (11.1%)

*CI* Confidence interval

**Fig. 1:**
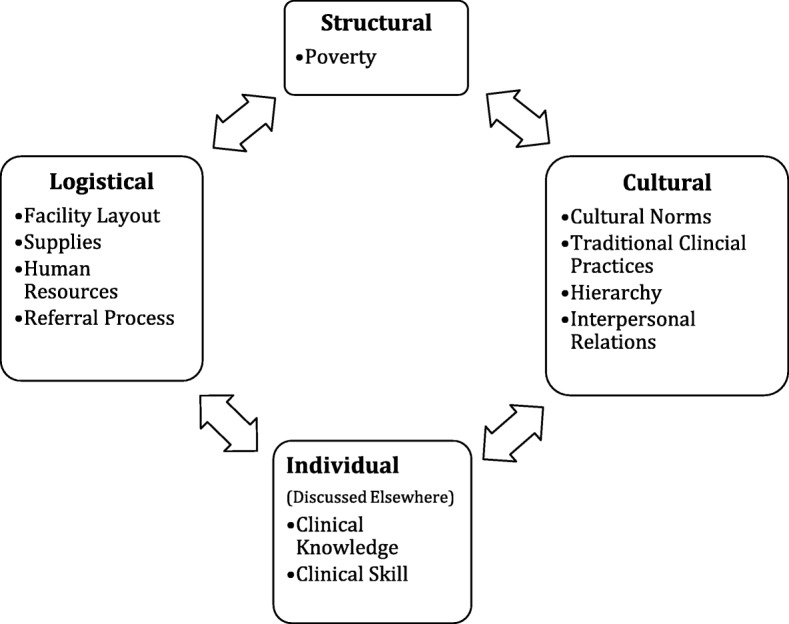
Summary of Barriers to Immediate Neonatal Care and Neonatal Resuscitation in Bihar

Codes were structured into a framework of four broad themes: logistical, cultural, structural, and individual barriers to EBP in immediate newborn care and NR (Fig. 1). Individual barriers are the topic of a separate manuscript [26].

### Logistical barriers

#### Facility layout

Mentors cited the distance between labor rooms and newborn care corners (NBCC), the spaces designated for NR in PHCs, as a barrier to ensuring an infant with poor or no respiratory effort was breathing or ventilated within the first minute after birth. Additionally, one mentor reported that one of the PHCs she worked in did not have a designated resuscitation space at all.

*"In this facility… we are not having [an] attached NBCC… It take[s] time to take the baby from the mother’s abdomen to the NBCC, so [mentees] are losing [the] golden minute..." (Age 24)*

*“Because of lack of infrastructure there is no different NBCC area, so it’s very difficult for [mentees] to do [resuscitations]." (Age 24)*

#### Supplies

Mentors unanimously agreed that supply issues including the availability, functionality, and accessibility of necessary resuscitation items, precluded the provision of effective immediate neonatal care and NR. Supplies most commonly cited as unavailable included mucus extractors, self-inflating ventilation bags, different mask sizes (particularly for preterm neonates), clocks (e.g. for measuring heart rate or timing NR), and oxygen. Additionally, mentors stated that due to resource constraints, towels, masks, and mucus extractors were often reused without being cleaned making them both less effective and an infection risk. Alternatively, families were asked to purchase these supplies outside the PHC creating delays in care and placing economic burdens on families. With the AMANAT intervention, mentors reported that the availability of ventilation bags improved.

*“In some facilities both mask [sizes are] also not there, like only one number mask is there, so if preterm baby is [born], it’s very difficult to give [bag mask ventilation].” (Age 24)*

*“Availability is not there in the PHC, so [families] go outside to get the mucus extractor or [mentees]… get the used one.” (Age 27)*

In addition, mentors reported that existing supplies were often not functional, e.g., due to leaks in ventilation bags or masks. Further, they reported PHCs often lacked reliable power supplies, which meant radiant warmers and oxygen concentrators could not be used when indicated.

*“Most of the times during [NR] actually radiant warmer is not working, oxygen concentrator is not working, ok their electronic devices are not working.” (Age 23)*

Finally, mentors explained that available supplies were often inaccessible as a result of disorganization in facilities, poor delivery preparedness, or improper staff handoffs between shifts.

*“Biggest barrier is there is a sister [i.e., mentee] in charge… she has the key of the main cupboard. In her duty, it is very easy to get the things. But the sister in charge [does not] hand over the keys to the… next staff… if any [asphyxiated infant] comes, the sister in charge might have given few things… we [must] have… the patients to buy from outside.”(Age unknown)*

#### Human resources

Almost all mentors felt that, given the workload at PHCs, there were an insufficient number of nurses on duty at any given time to deliver quality neonatal care.

*“Only two or three [mentees], they have to be doing everything… emergency condition they have to manage, they have to manage [operating theater] also, or family planning, and they have to manage in labor room also, and… routine immunization was also there… so it’s very hectic for them.” (Age 27)*

*“Only one [nurse] used to be there in [the] labor room and if baby did not cry, to whom will [she] see? To the mother or to the baby?” (Age 23)*

Additionally, mentors reported that doctors were frequently unavailable in PHCs. As mentees traditionally viewed birth asphyxia as the doctors’ responsibility, this created both a vacuum of providers to perform resuscitations and a lack of back-up support for mentees when they did learn to perform resuscitations.

*“[Mentees] have a preconception [that] to manage the baby [is] not their duty, it’s the duty of the doctor. Previously also when baby didn’t cry… they used to inform doctor ok baby is not crying… they do not try to be involved in the management of the baby." (Age 23)*

*“Doctors, every time doctors are not available in facilities, that’s the main thing, and most of the time these doctors won’t come to [the] labor room.” (Age 26)*

#### Referral process

Immediate referral of neonates from PHCs to district hospitals for a higher level of care was described as a common practice.

*"Doctors come…baby is very like bluish color, baby is convulsing… [they] say just refer the baby." (Age 27)*

Thus, mentors felt the logistical process of referring neonates was a significant barrier to immediate neonatal care. They explained that government ambulances were often unavailable, in which case families were asked to hire and pay for private transportation. Even in cases where ambulances were available, ambulance personnel had no medical training and were unable to continue resuscitation efforts during travel times ranging from 30 min to 2 h.

*"Most of the times ambulance is not available, so they [must take] baby in private vehicle." (Age 23)*

*“Can’t resuscitate in ambulance because in ambulance no medical person [is] with them to go to the referral hospital.” (Age 23)*

### Cultural barriers

#### Cultural norms

Early administration of oxytocin by traditional birth attendants outside of PHCs and late presentation of laboring mothers to PHCs were described as barriers to care in that they precipitated fetal distress and prevented early recognition of fetal distress respectively.

*“Over here the main issue is the mother comes only when she is fully dilated… as soon as she’s… on the entrance of delivery room she’s already [delivering]. And we are not able to check if the baby was in fetal distress… [only] after delivery we come to know." (Age unknown)*

After delivery, most mentors reported that mentees triaged appropriately when a non-vigorous neonate was born and required immediate attention. Nonetheless, mentors described cultural norms that influenced triage decisions and sometimes resulted in inadequate care of neonates. These norms included male infant preference and valuing the mother’s life over the infant’s.

*“I have seen a weak male baby is asphyxiated, [and] the family will be very in a very bad condition. And if the healthy female baby is asphyxiated, there would be no one around… I was carrying a female baby and begging in front of the attendant… ‘Please take the baby, please take the baby.’ Just because it was a female baby, no one cared." (Age unknown)*

*“If we said… this baby is going to die… [parents will say], if this dies, ok we’ll get another one… these thing[s] influence the [mentees].” (Age 25)*

*"Babies are not that much important here… actually their concept is like, if baby girl, means yeah, they can get another baby, but this mother’s life is much more important than that particular baby." (Age 25)*

Finally, one mentor explained that neonatal death was common enough in PHCs that nurses gradually became less concerned by it, and this negatively impacted care.

*"It happen[s], baby will die in hospital... so [mentees] are not taking that much worry." (Age 27)*

#### Traditional clinical practices

The majority of mentors felt that traditional clinical practices interfered with evidence-based immediate neonatal care. Mentors described practices including: holding the baby upside-down, immediately applying mustard seed oil, over stimulating the baby, waiting indefinitely for the baby to cry, and allowing female relatives currently breast feeding other children to immediately feed the neonate regardless of respiratory status.

*“And sometimes [mentees] will be roughing… like they will be… hyper-stimulating… and sometimes they will be keeping the baby up[side] down." (Age 28)*

*“They have mustard oil… for the oiling of the baby... whole body, head to toe of the baby… they used to tell that it provides heat… warmth to the baby.” (Age 27)*

*“[Mentees] used to be like… this is a normal thing… this will improve... we used to [say], we have to do something, baby is not crying, we should be active, but they used to be like… he will cry, no problem. Even half an hour… they don’t bother” (Age 23)*

According to mentors, the frequency of traditional practices decreased with PRONTO/AMANAT training. However, the use of EBPs represents a major clinical practice change and thus mentors felt it would require additional training and time to gain widespread acceptance and uptake.

*“Because they are doing from 30 years… 30 years [of old practices] and [only] 6 months [of training], [it] will take time.” (Age 27)*

#### Hierarchy

Mentors explained that hierarchy, with doctors and family members viewed as superior to nurses, dictated care in the delivery room and, in doing so, prevented mentees’ use of EBPs.

*“There was a baby [that] did not cry, and we told the [mentees] you do back rub, you do suction. Then we told them ok inform [doctor] also. [Doctor] came… he told them to take oil, back rub the baby, and… that baby died, died in front of us and [doctor] was blaming the mentees… However, doctor was giving wrong instructions… we cannot say to [doctor], ‘Ok you are telling the wrong thing.’” (Age 23)*

*“So it’s real scenario in Bihar… relatives they are… so much interrupting, ‘Ok what are you doing? Don’t do this, don’t do that.’”(Age 24)*

One mentor felt that the use of EBPs increased as PHC doctors, not directly included in training, were secondarily exposed to the AMANAT and PRONTO training of mentees. Finally, in addition to directly inhibiting the implementation of EBPs in resuscitations, mentors explained that hierarchy inhibited training mentees in EBPs due to competing priorities.

*“Administration support, like [doctors]… they want their immunization [rates to be] 100%, so they told… because of this training our immunization is disturbed. So it’s very challenging for us.” (Age 24)*

#### Interpersonal Relations

Almost every mentor mentioned interpersonal relations as a barrier to immediate neonatal care and NR in Bihar. The most frequently cited example was the relationship between mentees and patients’ families. Mentors explained that mentees feared physical and verbal abuse from patient relatives, as well as verbal abuse from delivering mothers when neonatal complications occurred. This fear deterred the provision of effective care.

*"[Mentees] are thinking if we are telling baby is having some problem… their family member is… maybe going to… beat them… so they are not telling mother… what happened with their babies. They are telling, ‘It’s ok, it’s ok, it’s becoming ok.’" (Age 27)*

*“[Mentees] were scared… scared of the public... they get threatening from the public… they say public comes in a jeep, many public, if any complications occur then they’ll come and they’ll shout." (Age 28)*

Mentors also reported that, driven by similar fears, doctors occasionally instructed the family members to visit an alternative facility, usually private, even if the baby had already died.

*"[Doctors] will say, ‘You go to private clinic and take your baby, baby is not well’… if baby is dead also [doctor] was telling… ‘Baby is alive, but you should take to another hospital.’ [Doctors felt that] if I’ll say… baby is dead, so they will do something to me. So [doctors] are giving false information to patient and patient relatives." (Age 24)*

Mentors additionally reported that the lack of restrictions on entry into the labor room resulted in many questions and commotion created by patients’ relatives, ultimately distracting nurses and interfering with their ability to provide effective care.

*"The entire village is in the labor room… so when [mentees] are explaining to one person, the other person… come[s] from [the] back and say[s], ‘What happened, what happened, why the baby is not crying, is everything ok?’… The nurse is not able to concentrate on the baby. [If] she makes [the] entire [group] to understand what is the scenario… in that only her whole energy goes.” (Age unknown)*

One mentor indicated that negative perceptions amongst local communities about the quality of care in PHCs contributed to this complicated relationship, but that the level of confidence in the quality of care improved as a result of PRONTO and AMANAT training.

*“Because previously… when the baby didn't cry, [mentees] used to [say], ‘Baby is ok, ok, ok’ and afterwards of 24 hours, the baby will die. So, family was like, ‘You only said that the baby's fine, now the baby [has] died.’ So now the families’ confidence is also increased that the nurse can do anything.” (Age 24)*

Finally, mentors felt that relationships amongst mentees themselves created barriers to effective neonatal care. Mentors reported that interpersonal conflicts frequently prevented effective teamwork amongst nurses in PHCs, which in turn negatively impacted the quality of neonatal care.

*“[Mentees] are saying that, ‘I’m senior and I’m having this much experience in field’, and the junior one is saying, ‘I’m junior, but in spite of all this I’m having experience of labor room.’ So, ego clashes." (Age 24)*

*"If she will call… [the other mentee will] ask, ‘Why I will come? This is your duty, so you have to do.’” (Age 24)*

One mentor described the practice of accepting money directly from the patients’ families as further contributing to the friction and interpersonal conflict between mentees.

*"[Mentees] are taking the money from the patients… if they are conducting the cases in their own time… If the nurse is having… two, three patients in her shift, so she doesn’t want to hand over… then she’ll be having problem in getting the money." (Age 27)*

### Structural barriers

#### Poverty

Poverty was felt to be an important overarching barrier to immediate neonatal care and NR. Mentors explained that poverty inhibited families from caring for ill neonates including their ability to follow referral recommendations. One mentor explained that this negatively impacted the standard of care for infants born to poorer families.

*"Even though we… explain [to] them your baby is going to die they will say, they will show like blank face, ‘Baby is going to die. We are poor. We cannot take that baby into that referral hospital. We cannot do anything for that baby. We are poor.’” (Age 25)*

*“If they are poor, parents won’t… get that much tense, means why should [mentees] take that much tension?” (Age 25)*

## Discussion

The provision of evidence-based immediate neonatal care and NR at PHCs in Bihar is hindered by numerous interwoven logistical, cultural, and structural barriers. Together, these barriers affect every aspect of immediate newborn care. Cultural norms including early administration of oxytocin and delayed presentation of laboring mothers to PHCs, precipitate and delay identification of fetal distress respectively. Distance between labor rooms and resuscitation areas prohibits timely initiation of NR. Supply issues, traditional clinical practices, and hierarchy prevent performance of key EBPs in NR such as bag mask ventilation. Finally, poverty and ineffective referral systems contribute to inadequate clinical care and unsafe transfers for the sickest neonates.

Key themes in this manuscript, including facility infrastructure and resources, referral processes, cultural norms, traditional practices, hierarchy, interpersonal relations, and poverty have been previously described as common barriers to neonatal care in LMICs in multi-country analyses [7–9, 11] and in qualitative evaluations of the Helping Babies Breathe program [27, 28]. However, to our knowledge, this is the first in-depth characterization of these barriers in Bihar. We believe this more nuanced understanding of the specific issues that sum into these larger thematic barriers will be key to addressing them. While we recognize solutions are not simple, we propose the following key action items.

### Logistical barriers and action items

Logistical barriers identified by mentors included poor facility infrastructure, inadequate supplies, human resource shortages, and failures in the referral process. Regarding facility infrastructure, the majority of mentors explained that long distances between labor rooms and resuscitation areas prevented timely initiation of resuscitations. This issue of proximity of NBCCs has been previously cited as a barrier to care in Bihar [15], and is distinct from lacking a resuscitation space all together-- a barrier identified in Tanzania in conjunction with the Helping Babies Breathe program’s emphasis on ‘the golden minute’ [27]. Priority should be placed on moving dedicated resuscitation areas into labor rooms to enable mentees to establish effective ventilation within the first minute of life.

Like many LMICs, the availability of resuscitation supplies has also been identified as a barrier to care in Bihar [15]. However, mentors in this study additionally identified functionality and accessibility of already available supplies as equally important issues. While ensuring the consistent availability of supplies such as mucus extractors, preterm-size masks, and clean towels is essential, training providers on delivery preparedness and handover techniques may improve the accessibility and timely utilization of already available equipment [28]. Moreover, ensuring a more reliable power supply at PHCs would facilitate use of available radiant warmers and oxygen concentrators when indicated. However, training should continue to emphasize the importance of ventilation with room air using self-inflating bags and kangaroo care so that power supply does not become an unnecessary or false barrier to care.

Provider shortages been previously indentified as a problem in Bihar based on demographic data [17]. Mentors unanimously felt the number of ANMs/GNMs was insufficient for the volume at PHCs and left nurses with difficult decisions about who to care for should maternal and neonatal emergencies co-exist. Although not explicitly mentioned by mentors in interviews, a recent systematic review identified workload as a strong contributor to provider burn out and emotional fatigue in LMICs [29], which undoubtedly effects quality of care. While, training more ANMs and GNMs should remain a long-term goal [30], a more immediate solution may be thoughtful task shifting within PHCs to less skilled providers including community health workers known as Ashas [31]. Approximately 10% of neonates require only the initial steps of NR (drying, warming, and stimulating) whereas 3–6% require further intervention including bag-mask ventilation [32]. Ashas could be trained to provide the initial steps of NR with clear guidance to immediately alert an ANM/GNM if a neonate does not transition with initial resuscitation measures. In turn, ANMs/GNMs could shift their focus to more complicated deliveries or other PHC duties, which may be particularly valuable in high-volume facilities.

Finally regarding referrals, future clinical training should teach indications for referral and measures to stabilize neonates who require more than basic resuscitation prior to transfer. However, given the reality of resource and personnel limitations at PHCs, improving the referral process will be key to improving neonatal outcomes. With the increase in hospital-based births in India, previous research has called attention to the importance of a well-linked referral system for neonates [33]. Mentors identified the following key action items: eliminating the financial barrier for families to referral by providing free government transport and providing basic NR training to transport personnel so that resuscitation efforts can continue if necessary. In Madhya Pradesh, there is ongoing work to bolster the referral system that may serve as an example [34].

### Cultural barriers and action items

Cultural barriers indentified by mentors included norms, traditional clinical practices, hierarchy, and interpersonal relations. Regarding cultural norms, demographic data from Bihar has demonstrated decreased survival among female infants relative to male infants [22], supporting what mentors cited as male infant preference. Addressing this and the perceived value of an infant’s life compared to that of the mother, particularly when obstetric and neonatal complications occur simultaneously, requires further research to better understand local values. Nonetheless, inappropriate administration of oxytocin, which has been proven to increase the risk of perinatal death in rural Bangladesh [35], and late maternal presentation to PHCs, which prevents fetal monitoring that has been identified as a key determinant of resuscitations in Tanzania [28], may be more easily addressed with birth planning during antenatal time.

The acceptance of EBPs in place of traditional clinical practices may be facilitated by ongoing exposure of all levels of providers, from Ashas to doctors, to demonstrate the effectiveness of EBPs in real deliveries. The inclusion of doctors in future trainings at PHCs should also be a priority to ensure that providers across all levels of care are aware and knowledgeable about current clinical guidelines to limit hierarchical conflicts. This may also improve the degree to which mentees feel supported in their clinical practice, another factor identified as being protective against burn out and emotional fatigue [29].

In PHCs in Bihar, the relationship between nurses and patients’ relatives has a large impact on quality of care, as mentees routinely fear physical and/or verbal harm from delivering woman and their relatives. Implementing standard labor room limits on the number of family members permitted to enter at one time, may promote a more secure work environment and, in turn, improve neonatal care. Emphasizing communication and team training in any clinical skills training, which has been proven to be effective in PRONTO training in Mexico [36], may also empower ANMs/GNMs to better navigate both relations with difficult families and tense interpersonal relations amongst themselves.

### Structural barriers and action items

Poverty was described as an overarching structural barrier to immediate neonatal care and NR. While there is no clear solution to this systemic problem, improved equality of care may be achieved by eliminating out-of-pocket expenditures by families in public facilities for essential resuscitation equipment and for transportation to referral facilities. These financial burdens continue to exist despite the Janani Shishu Sukaksha Karyakaram program launched by the Government of India in 2011 to eliminate out-of-pocket costs for maternal and neonatal care at public facilities [37]. Additionally, any clinical skills training should be respectful of cultural values yet create a standard of care and emphasize its relevance to all infants regardless of their family’s economic status.

### Limitations

This study has several limitations. First, it does not provide an exhaustive characterization of all barriers to the provision of evidence-based immediate newborn care and NR in Bihar. It intentionally excludes barriers related to individual providers, such as clinical knowledge and skills, which are discussed in another manuscript [26]. Additionally, although the mentors interviewed in this study had experience mentoring in PHCs, which included a combination of direct clinical care and supervision of clinical care, they were all from other states in India. This may have introduced information bias if interviewees had preconceptions about Bihar or local healthcare providers. To reduce the likelihood of this bias, mentors were only eligible for interview if they had at least 16 months of experience working in eight different PHCs. Additionally, interviews were conducted by a member of the United States based research team which, could have introduced interpretation bias. To mitigate this risk, interviews were conducted with fluent English speaking mentors and the same individual who conducted all interviews transcribed and analyzed all data. Double coding of two transcripts by another member of the research team demonstrated consistency across all themes, thus the overall magnitude of the aforementioned biases was likely minimal.

## Next steps and conclusion

Further study of barriers to immediate neonatal care and resuscitation in Bihar including interviews with ANMs and GNMs, doctors, and community members would be useful for data triangulation and validation. It would additionally provide a more holistic view of barriers to care to guide future interventions. Nevertheless, in an effort to maximize the benefit of ongoing training, the next iteration of the PRONTO curriculum in Bihar has already been adapted to address some of the action items discussed above. Examples include incorporating training on delivery preparedness and provider handover techniques to improve effective utilization of supplies, as well as strengthened inter-professional and team training to address hierarchical issues between doctors and mentees and dynamics between mentees themselves.

This study has also identified barriers such as human resource shortages, ineffective referral systems, and challenging relationship dynamics between nurses, delivering mothers, and their families, which cannot be addressed by clinical training programs alone. Rather, addressing these barriers likely requires commitment from local government, strong local partnerships, and community outreach. Nevertheless, an understanding of such barriers is an essential beginning and important for adapting skills-based trainings developed in Western contexts to settings such as Bihar with the aim of reducing neonatal mortality in India to the SDG target by 2030 [3, 5, 6].

## Abbreviations

ANM: Auxiliary nurse midwife
CI: Confidence interval
EBP: Evidence-based practices
GNM: General nursing and midwifery
LMIC: Low- and middle-income countries
NBCC: Newborn care corner
NMR: Neonatal mortality rate
NR: Neonatal resuscitation
PHC: Primary health center
SDG: Sustainable Development Goals
